# Surface settlement induced by frictional force of epb shield tunneling in sandy gravels

**DOI:** 10.1371/journal.pone.0310111

**Published:** 2024-09-10

**Authors:** Xuening Rong, Lirong Gao, Chaoling Ji, Aimin Han, Hailong Liu, Songtong Han

**Affiliations:** 1 College of Transportation Engineering, Nanjing Tech University, Nanjing, China; 2 Department of Civil Engineering, Dalian Maritime University, Dalian, China; 3 Heilongjiang North Tools Co., Ltd, Mudanjiang, China; Guizhou University, CHINA

## Abstract

The excavation of Earth Pressure Balance (EPB) shield can be divided into two distinct stages, i.e. advancing and lining installation. The frictional force applied on surrounding soils reverses at these two stages, which is harmful to the settlement control. Based on Mindlin’s method, a new model of surface settlement is derived to involve the reversed friction. A closed form formula is then obtained for the major type of metro tunnels. Main operational parameters are also used as input of the formula. Numerous operational data and measured settlements are collected from EPB tunnels of Chengdu Metro, Line 7. The proposed formula is validated against these field data in sandy gravels. It is shown that the new formula gives reasonable prediction of surface settlement along the tunnel sections. The accuracy of new formula is significantly higher than that of Peck’s formula. This study provides a new vision in settlement control of EPB shield tunneling. The increase of chamber pressure will induce higher negative friction during the lining installation. Therefore, surface settlement of EPB tunneling cannot be controlled by just increasing chamber pressure. A balanced relationship between the chamber pressure and the thrust should be maintained instead.

## 1. Introduction

In urban areas, ground settlement induced by shield tunneling inevitably disturb surrounding roads, buildings, and pipeline facilities [1]. The prediction and control of ground settlement is one of the key issues in tunnel construction. In engineering practice, the most commonly used method for settlement prediction is an empirical approach based on Peck’s formula [2–4]. Peck’s formula predicts ground settlement with empirical parameters such as the ratio of ground volume loss (*V*_*lr*_). For varied geological conditions, empirical values of *V*_*lr*_ have been reported in the literatures [5–7], thus the surface settlement can be calculated by a simple formula. Peck’s formula does not include specific information of the shield excavation, and is mainly used to estimate the average settlements along entire tunnel section. Without any operational parameter, Peck’s formula is not useful for the settlement control of EPB shield.

To include real operational parameters, settlement prediction based on analytical or numerical models have been developed [7–11]. These models often incorporate major operational parameters into a mechanical process of excavation [12]. Data-driven model, i.e. machine learning or other statistical algorithms [13–15], has also been used in the prediction of ground movement [16–18]. It has been revealed that operational parameters such as chamber pressure, thrust etc., play very important role in the settlement control [9–11]. According to existing theories, higher chamber pressure tends to uplift the soil in front of cutterhead, and thus reduces the surface settlement [2]. Based on these theories, it was suggested that the surface settlement can be reduced by increasing the chamber pressure [19]. In the field measurements, however, it was also observed that the surface settlement increases with higher chamber pressure [20,21]. Compared to the real excavation of EPB shield, the existing theories are quite simplified. This simplification creates uncertainty in the relationship between surface settlement and the operational parameters. Therefore, the adjustment of operational parameters still relies on the instinct of shield drivers.

Theoretical analyses of tunnel induced settlement mainly involve the Mindlin integral [22–24] and the stochastic medium method [25,26]. These analyses assume that the EPB shield advances at a uniform speed during the entire excavation process. This means the EPB machine stays in the same state of stress equilibrium. But in the real EPB excavation, there are two distinct stages, i.e. advancing and lining installation. The loading conditions during these two states are significantly different. The existing analytical models mainly focus on the first stage of advancing. In this stage, the frictional force applied on surrounding soils is in the same direction with EPB advancement. During the stage of lining installation, however, the thrust of tail jack is usually reduced, leading to a negative frictional force around the shield machine. The effect of negative frictional force has not been considered in theoretical models. This may lead to systematic errors in the prediction of surface settlement.

To improve the analytical method of surface settlement, this study derived a new model which considers the friction during different stages. For the EPB machines used in urban metro, the integral formulas were simplified into a closed form formula for fast estimation of surface settlement. The new formula was then validated against the field measurements in sandy gravels of Chengdu Metro Line 7. Compared to Peck’s formula, the new formula gives more reasonable prediction of surface settlement. With consideration of real operational parameters, the new formula is also more useful in the settlement control.

## 2. Analysis of surface settlement with negative frictional force

### 2.1. Variation of thrust and frictional force during advancing and lining installation

To analyze the interaction between the shield machine and surrounding soils, the excavation of shield tunnel is generally simplified as a continuous advancement. The jack thrust and shield-soil friction are assumed to be constant [23,27]. However, for actual shield tunnels, the excavation process is divided into two distinct stages:

1. Advancing stage: The shield machine maintains an excavation speed of approximately 50 mm/min under the jack thrust. The advancing is continuous until the distance reaches the length of a single lining (approximately 1.5 m);
2. Lining installation stage: The shield machine remains stationary for lining installation. The jack thrust is often set to zero for the convenience of installation. The soil chamber pressure is basically constant (none zero).

**Fig 1:**
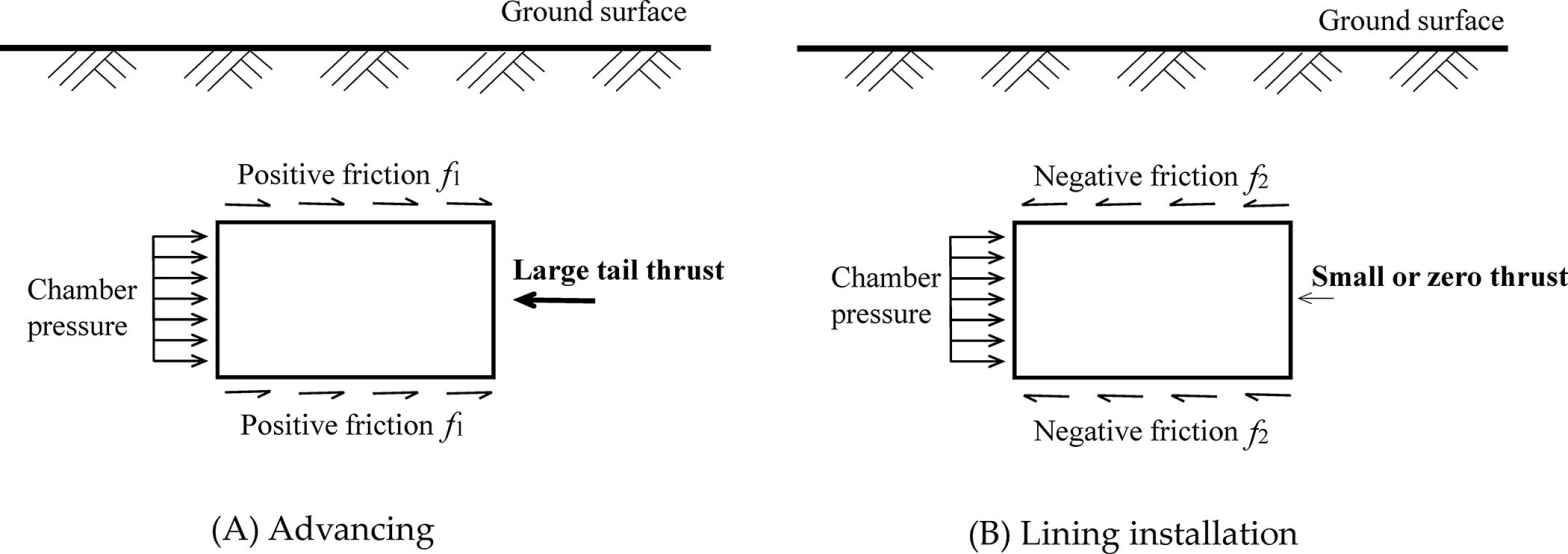
Balance analysis of shield machine at different stages. (A) Advancing. (B) Lining installation.

The stress analysis during advancing and lining installation are shown in Fig 1, respectively. From Fig 1A, the force equilibrium during the shield advancing can be derived as follow:

$$2\pi RLf_{1}+\pi R^{2}p_{c}=T$$ (1)

where: *f*_1_ (kPa) is the positive frictional force along the shield surface; *p*_*c*_ is the face pressure at the cutterhead (kPa); *T* (kN) is the jack thrust; *R* (m) is the radius of the cutterhead; *L* (m) is the shield length. In Eq (1), the operational parameters *p*_*c*_ and *T* can be measured directly by the sensors of shield machine. The only unknown variable is the positive frictional force *f*_1_, which can be obtained by the other parameters as follow:

$$f_{1}=\frac{T-\pi R^{2}p_{c}}{2\pi RL}$$ (2)

During the stage of lining installation, as shown in Fig 1B, the thrust of tail jacks become zero. The force equilibrium of the shield machine can be derived as follow:

$$2\pi RLf_{2}+\pi R^{2}p_{c}=0$$ (3)

where: *f*_2_ (kPa) is the negative frictional force during lining installation. Based on other known parameters, the value of the negative frictional force during the lining installation can also be obtained:

$$f_{2}=\frac{-p_{c}R}{2L}$$ (4)

**Fig 2:**
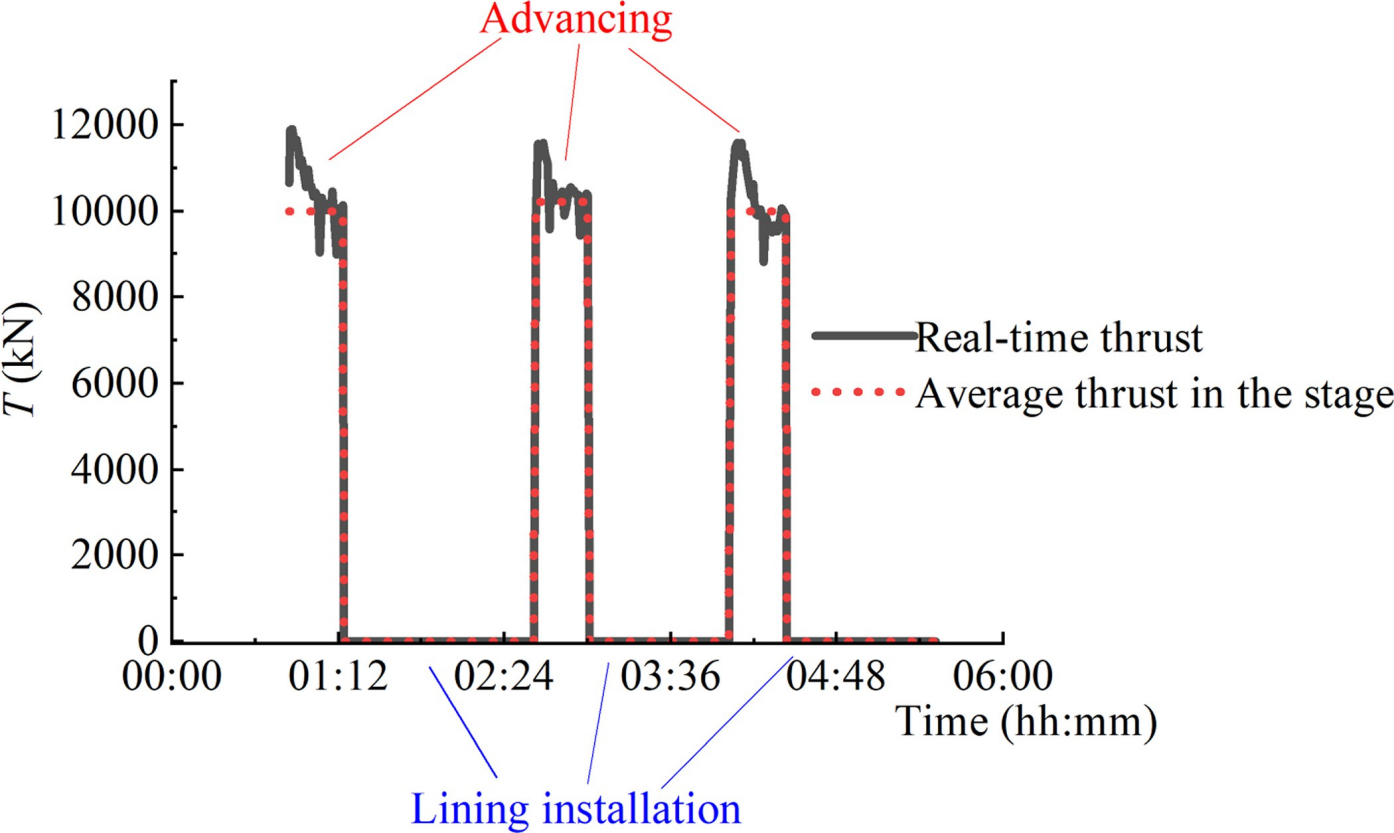
Variation of thrust during different stages of excavation.

**Fig 3:**
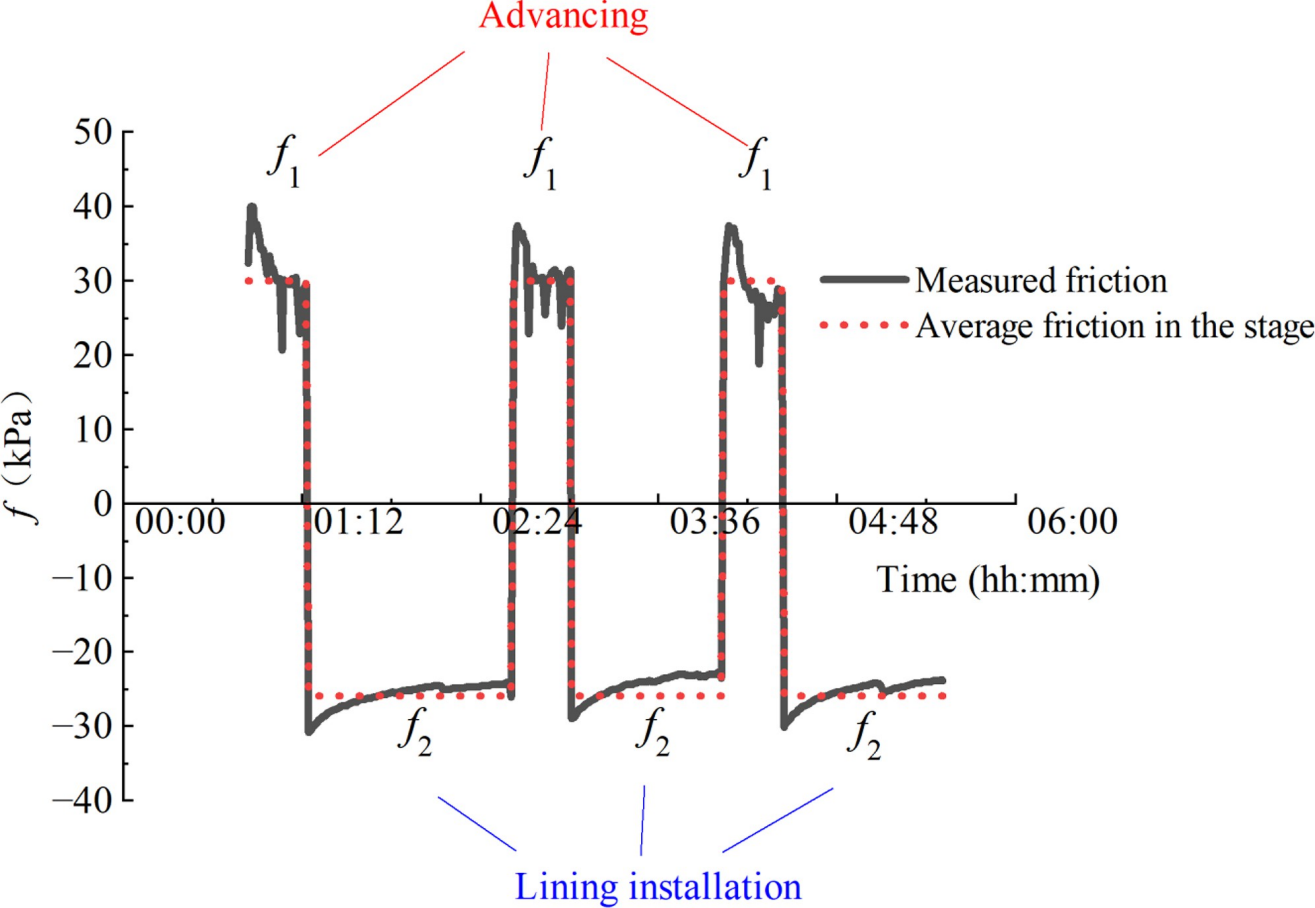
Variation of frictional force during different stages of excavation.

During the excavation process, the stage of advancing and lining installation changes repeatedly. The jack thrust fluctuates between positive thrust and zero, while the shield-soil friction changes between positive values and negative ones. Fig 2 shows the real-time thrust recorded by a shield machine of Chengdu Metro Line 7. It can be observed that the measured thrust varies between 0 and 10400 kN for these adjacent rings. Based on the measurement of thrust and chamber pressure, the shield-soil friction can be calculated from Eqs (2) and (4). The result of calculation is shown in Fig 3. It can be seen that the frictional force changes between -26 kPa and 30 kPa. During the lining installation, the shield-soil friction is negative. The value of the negative friction is mainly determined by the soil chamber pressure and the size of the shield machine.

### 2.2. Surface settlement induced by variation of shield-soil friction

**Fig 4:**
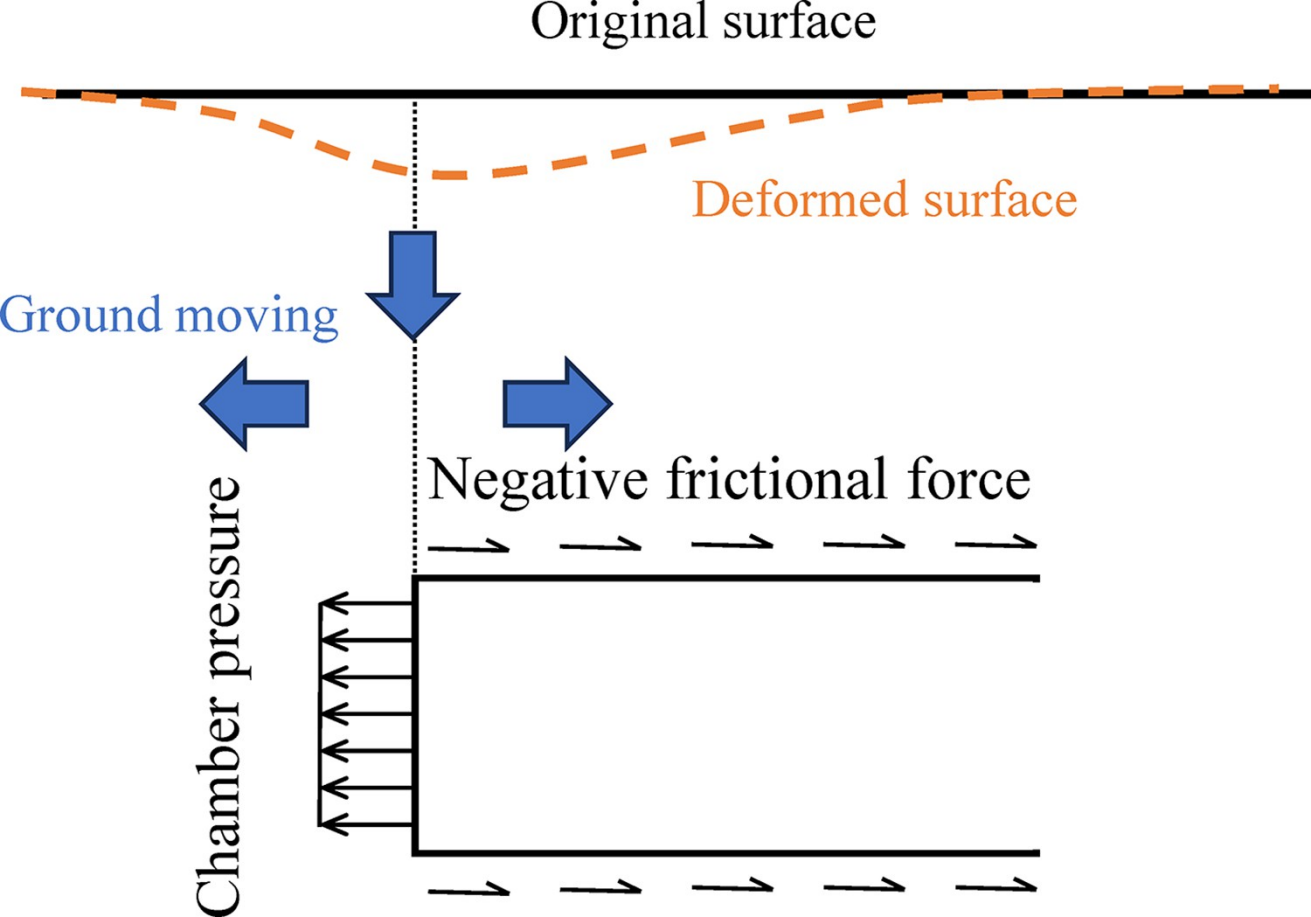
Surface settlement induced by negative frictional force.

For the two different stages of shield tunneling, the direction of shield-soil friction is totally opposite. The ground deformation caused by positive frictional force during the shield advancing has been analyzed based on Mindlin’s solution [23,24,27]. While the effect of negative friction during the lining installation was ignored. During the advancing stage, the positive frictional force tends to uplift the soils above the shield machine [19,27]. The displacement of uplift can be calculated by integration of Mindlin’s solution. During lining installation, however, the negative frictional force will cause settlement of the ground surface. The principle of surface settlement induced by negative friction is shown in Fig 4. Because the soil tends to move separately away from the cutterhead, the ground surface moves down to fill the void.

Based on Mindlin’s solution, the settlement of ground surface induced by shield-soil friction can be derived as follow [27,28]:

$$w_{f}=\frac{fR}{4\pi G}\int_{0}^{2\pi }\int_{0}^{L}\left(x+l\right)[\frac{-h+R\sin\theta }{W_{1}^{3}}+\frac{\left(1-2\mu \right)}{W_{1}(W_{1}+h-R\sin\theta )}]dld\theta$$ (5)

where: *w*_*f*_ (mm) is the settlement of ground surface induced by shield-soil friction; *f* (kPa) is the lateral frictional force applying on the soils that surround the shield machine, with positive values during the advancing stage; *x* denotes the horizontal distance of the ground surface from the cutterhead; *G* and *μ* is the shear modulus and Poisson’s ratio of ground soil, respectively; *R* and *L* (in m) is the radius and the length of shield machine, respectively; *h* (m) is the depth of the tunnel axis; *W*_1_ (m) is a combined parameter for convenience of the calculation, which is determined by the location and the size of shield machine as $W_{1}=\sqrt{{\left(x+l\right)}^{2}+h^{2}+R^{2}-2Rh\sin\theta }$.

By substituting Eqs (2) into (5), the ground settlement caused by positive frictional force (*w*_*f*1_) can be obtained as follow:

$$w_{f1}=\frac{T-\pi R^{2}p_{c}}{8\pi^{2}GR}\int_{0}^{2\pi }\int_{0}^{L}\left(x+l\right)[\frac{-h+R\sin\theta }{W_{1}^{3}}+\frac{\left(1-2\mu \right)}{W_{1}(W_{1}+h-R\sin\theta )}]dld\theta$$ (6)

Similarly, by substituting Eqs (4) into (5), the ground settlement caused by negative frictional force (*w*_*f*2_) can be obtained:

$$w_{f2}=\frac{-Rp_{c}}{8\pi G}\int_{0}^{2\pi }\int_{0}^{L}\left(x+l\right)[\frac{-h+R\sin\theta }{W_{1}^{3}}+\frac{\left(1-2\mu \right)}{W_{1}(W_{1}+h-R\sin\theta )}]dld\theta$$ (7)

Combining Eqs (6) and (7), the total settlement *w*_*f*12_ induced by the shield-soil friction can be obtained as follow:

$$w_{f12}=\frac{T-2\pi R^{2}p_{c}}{8\pi^{2}GR}\int_{0}^{2\pi }\int_{0}^{L}\left(x+l\right)[\frac{-h+R\sin\theta }{W_{1}^{3}}+\frac{\left(1-2\mu \right)}{W_{1}(W_{1}+h-R\sin\theta )}]dld\theta$$ (8)

Meanwhile, the value of *w*_*f*12_ is just one component in total surface settlement. There are two other components caused by additional face pressure *p* and the ground volume loss *V*_*loss*_, respectively [22,27,28]. Based on Mindlin solution, the integral expression for surface settlement induced by additional face pressure *p* can be obtained as follow [27]:

$$w_{p}=\frac{px}{4\pi G}\int_{0}^{2\pi }\int_{0}^{R}[\frac{-h+r\sin\theta }{W_{2}^{3}}+\frac{1-2\mu }{W_{2}(W_{2}+h-r\sin\theta )}]rdrd\theta$$ (9)

where: *w*_*p*_ (mm) is the surface settlement caused by additional face pressure; *W*_2_ (m) is a combined parameter determined by the location as $W_{2}=\sqrt{x^{2}+h^{2}+r^{2}-2rh\sin\theta }$, which is used for convenience of the calculation.

Another component of surface settlement is induced by ground volume loss of the tail gap. The surface settlement induced by ground volume loss can be calculated using the following equation [27,29]:

$$w_{l}=\frac{V_{\mathrm{loss}}}{2\pi h}$$ (10)

where, *w*_*l*_ (mm) is the surface settlement induced by ground volume loss; *V*_*loss*_ (m^2^) is the volume of ground volume loss per unit meter of advancement. *V*_*loss*_ can be estimated from the width of tail gap *δ*. Note that *δ* is actually the sum of shell thickness and lining installation gap. Because *δ* is much smaller than the tunnel diameter, *V*_*loss*_ = 2*πRδ*.

The total settlement induced by shield excavation can be obtained by the sum of three components as follow:

$$\begin{array}{l} w=w_{f12}+w_{p}+w_{l}\\ \;=\frac{T-2\pi R^{2}p_{c}}{8\pi^{2}GR}\int_{0}^{2\pi }\int_{0}^{L}\left(x+l\right)[\frac{-h+R\sin\theta }{W_{1}^{3}}+\frac{\left(1-2\mu \right)}{W_{1}(W_{1}+h-R\sin\theta )}]dld\theta +\\ \;\frac{px}{4\pi G}\int_{0}^{2\pi }\int_{0}^{R}[\frac{-h+r\sin\theta }{W_{2}^{3}}+\frac{1-2\mu }{W_{2}(W_{2}+h-r\sin\theta )}]rdrd\theta +\frac{V_{\mathrm{loss}}}{2\pi h} \end{array}$$ (11)

Because of the shield and lining structure, the settlement can be calculated right above the cutterhead. Thus, Eq (11) can be simplified by *x* = 0 as follow:

$$w=\frac{T-2\pi R^{2}p_{c}}{8\pi^{2}GR}\int_{0}^{2\pi }\int_{0}^{L}[\frac{-h+R\sin\theta }{W_{1}^{3}}+\frac{\left(1-2\mu \right)}{W_{1}(W_{1}+h-R\sin\theta )}]ldld\theta +\frac{V_{\mathrm{loss}}}{2\pi h}$$ (12)

Eq (12) is the integral expression for surface settlement involving the actual shield-soil friction. Eq (12) is appropriate for sand or gravel stratum with high permeability, because the deformation converges rapidly in these soils. However, the expression does not consider subsequent creep deformation, and it is not suitable for saturated clay with low permeability.

### 2.3. Solution and simplification of the settlement formula

The Eq (12) of surface settlement cannot be integrated directly, thus it is not convenient in engineering practice. Based on a large amount of numerical integrations, a simplified method is developed in this study. A closed-form expression is finally derived for settlement calculation.

Dimensional analysis reveals that the result of double integration in Eq (12) is a dimensionless number, which can be denoted as *β*_*h*_:

$$\beta_{h}=\int_{0}^{2\pi }\int_{0}^{L}[\frac{-h+R\sin\theta }{W_{1}^{3}}+\frac{\left(1-2\mu \right)}{W_{1}(W_{1}+h-R\sin\theta )}]ldld\theta$$ (13)

where the coefficient *β*_*h*_ is the frictional settlement coefficient, indicating the proportional relationship between shield-soil friction and ground settlement. *β*_*h*_ is mainly determined by the tunnel depth and the dimensions *R* and *L* of shield machine.

**Fig 5:**
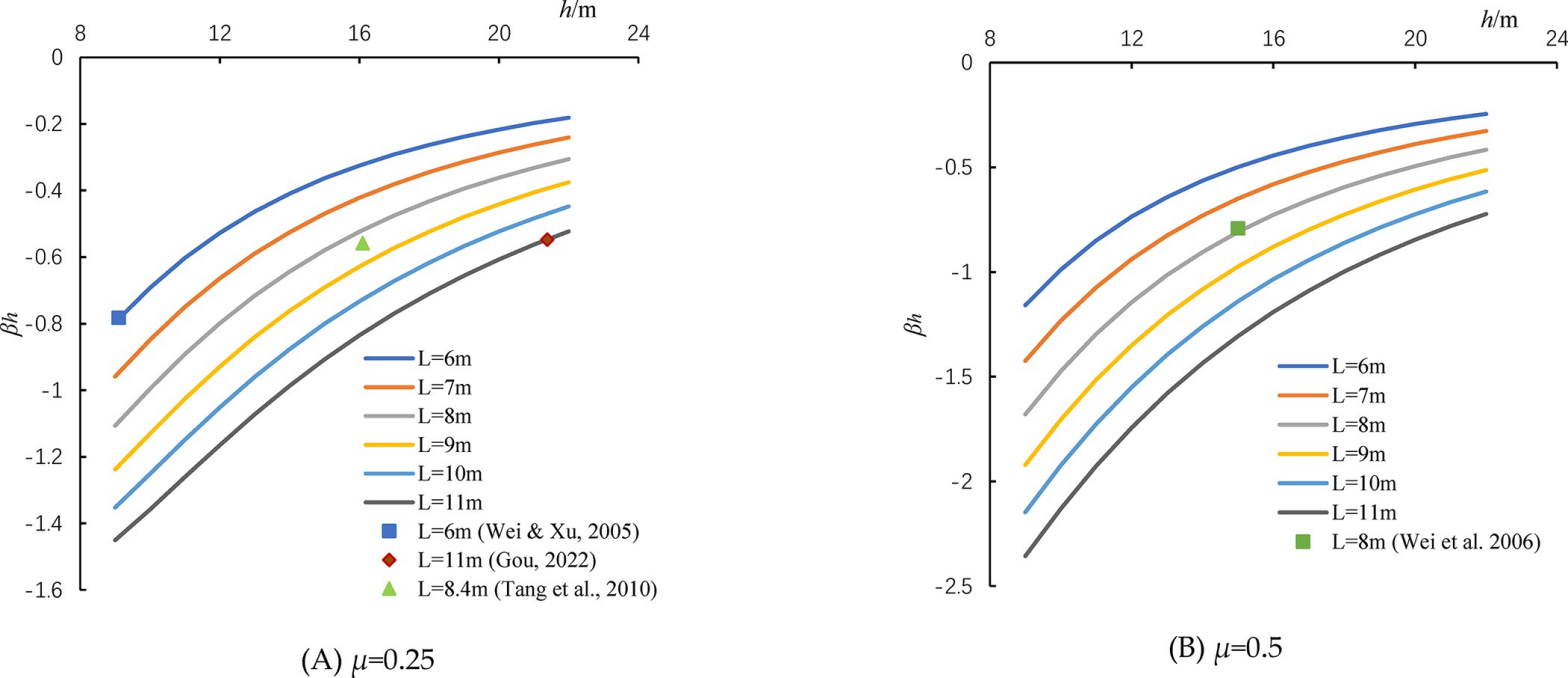
Relationship between the frictional settlement coefficient *β*_*h*_ and the shield length *L*, tunnel depth *h*. (A) *μ* = 0.25. (B) *μ* = 0.50.

For real metro tunnels, a uniform cross-section is often adopted. For most metro tunnels in China, the radius *R* is a constant value of 3.14 m, while the shield length *L* may vary. For a specific EPB tunnel, however, both the shield radius *R* and length *L* are constant, thus the coefficient *β*_*h*_ is only determined by the tunnel depth *h*. Based on Legendre-Gauss quadrature [27,28], the values of frictional settlement coefficient for different shield lengths and tunnel depths can be calculated from Eq (13). The results of calculation are shown in Fig 5. The figure is divided into two parts, Fig 5A and 5B, based on the different value of Poisson’s ratio *μ*. It can be observed that the frictional settlement coefficients are negative, indicating that the positive frictional force induces uplift displacement (negative settlement), while negative frictional force induces settlement. For a specific shield machine, the absolute value of *β*_*h*_ decreases with increasing tunnel depth. The relationship between *β*_*h*_ and *h* is very similar to hyperbolic curves, implying that deeper excavation has a smaller influence on surface settlement. With an increase in shield length *L*, the curve of *β*_*h*_ shifts downward because the contact area applied by frictional force increases. This means a greater influence on surface settlement.

As shown in Fig 5, the obtained curves of *β*_*h*_ are also compared with that reported in the literatures [27,28,30,31]. In previous studies, the settlement induced by frictional force is calculated at a fixed tunnel depth *h*, thus the results are represented as scattered points. It can be seen that the reported values of *β*_*h*_ are coincident with the curves obtained in this study. If the shield length is an integer (*L* = 6, 8, 11 m), the reported values of *β*_*h*_ are just on the curves [27,28,31]. If the shield length is not an integer (*L* = 8.4 m) [30], the value of *β*_*h*_ locates between two adjacent curves (*L* = 8m and *L* = 9m). Therefore, the frictional settlement coefficients obtained in this study for various shield lengths and tunnel depths are reliable.

For a certain shield machine, it is also observed that the frictional settlement coefficient *β*_*h*_ is inversely proportional to the tunnel depth. Therefore, the expression of *β*_*h*_ can be simplified as a hyperbolic function as follow:

$$\beta_{h}=-\frac{a}{h}+b$$ (14)

where the parameters *a* and *b* can be determined by the regression analysis based on the data shown in Fig 5. For varied Poisson’s ratios and shield lengths, the obtained values of *a* and *b* are shown in Table 1. The R-squared (*R*^2^) values for all the regression analysis are very close to unity. Therefore, the error induced by replacing the integral formula with the hyperbolic function (i.e. Eq 14) is negligible.

**Table 1:** Values of *a* and *b* in Eq (1,4).

*μ*		*L* (m)	*a* (m)	*b*		*R* ^2^	
0.25	6		9.464		0.2600		0.9991
	7		11.154		0.2711		0.9996
	8		12.535		0.2598		0.9984
	9		13.588		0.2270		0.9955
	10		14.318		0.1748		0.9911
	11		14.745		0.1059		0.9852
0.3	6		10.354		0.2905		0.9987
	7		12.292		0.3088		0.9997
	8		13.927		0.3043		0.9991
	9		15.234		0.2778		0.9972
	10		16.215		0.231		0.9941
	11		16.889		0.1668		0.9899
0.35	6		11.245		0.3209		0.9983
	7		13.429		0.3465		0.9996
	8		15.318		0.3489		09995
	9		16.88		0.3285		0.9983
	10		18.113		0.2873		0.996
	11		19.033		0.2278		0.993
0.4	6		12.135		0.3514		0.9979
	7		14.567		0.3842		0.9994
	8		16.709		0.3934		0.9997
	9		18.526		0.3793		0.9989
	10		20.011		0.3436		0.9973
	11		21.177		0.2888		0.995
0.45	6		13.026		0.3818		0.9976
	7		15.705		0.4219		0.9992
	8		18.1		0.438		0.9997
	9		20.172		0.4301		0.9994
	10		21.908		0.3999		0.9982
	11		23.155		0.3427		0.9966
0.5	6		13.917		0.4123		0.9972
	7		16.843		0.4596		0.9990
	8		19.492		0.4823		0.9997
	9		21.817		0.4809		0.9996
	10		23.806		0.4562		0.9988
	11		25.465		0.4107		0.9974

By substituting Eqs (14) and (13) into Eq (12), the calculation of surface settlement can be simplified as follow:

$$\begin{array}{l} w=\frac{T-2\pi R^{2}p_{c}}{8\pi^{2}GR}\beta_{h}+\frac{V_{\mathrm{loss}}}{2\pi h}\\ \;=\frac{T-2\pi R^{2}p_{c}}{8\pi^{2}GR}(-\frac{a}{h}+b)+\frac{V_{\mathrm{loss}}}{2\pi h} \end{array}$$ (15)

In engineering practice, the values of *a* and *b* can be obtained from Table 1. If the shield length *L* is non-integer, values of *a* and *b* can be determined by interpolation. Eq (15) provides a closed form formula for surface settlement. By including Eqs (6) and (7), Eq (15) considers the settlement caused by both the positive and negative friction. It can be used to predict the ground settlement with specific operational parameters, such as thrust and chamber pressure. It is also more convenient than existing integral formulas.

With a negative value of *β*_*h*_, it can be seen in Eq (15) that an increase in chamber pressure will induce greater surface settlement. This phenomenon is not reported in traditional theories [19], because the negative frictional force during the lining installation is usually neglected. Actually, the chamber pressure should be balanced by negative frictional force during the lining installation. Higher *p*_*c*_ leads to larger negative frictional force around the shield machine, and thus results in greater surface settlement. The positive correlation between the surface settlement and chamber pressure is observed in the field measurements [20]. In the shield tunneling of Changzhou and Chengdu Metro, positive correlation between the surface settlement and chamber pressure is also observed. These observations cannot be explained by traditional theory of EPB tunneling, but it is consistent with the settlement analysis considering negative frictional force. In the next section, this new model is validated against the field data obtained from the shield tunneling of Chengdu Metro, Line 7.

## 3. Case analysis

### 3.1. Engineering background

**Fig 6:**
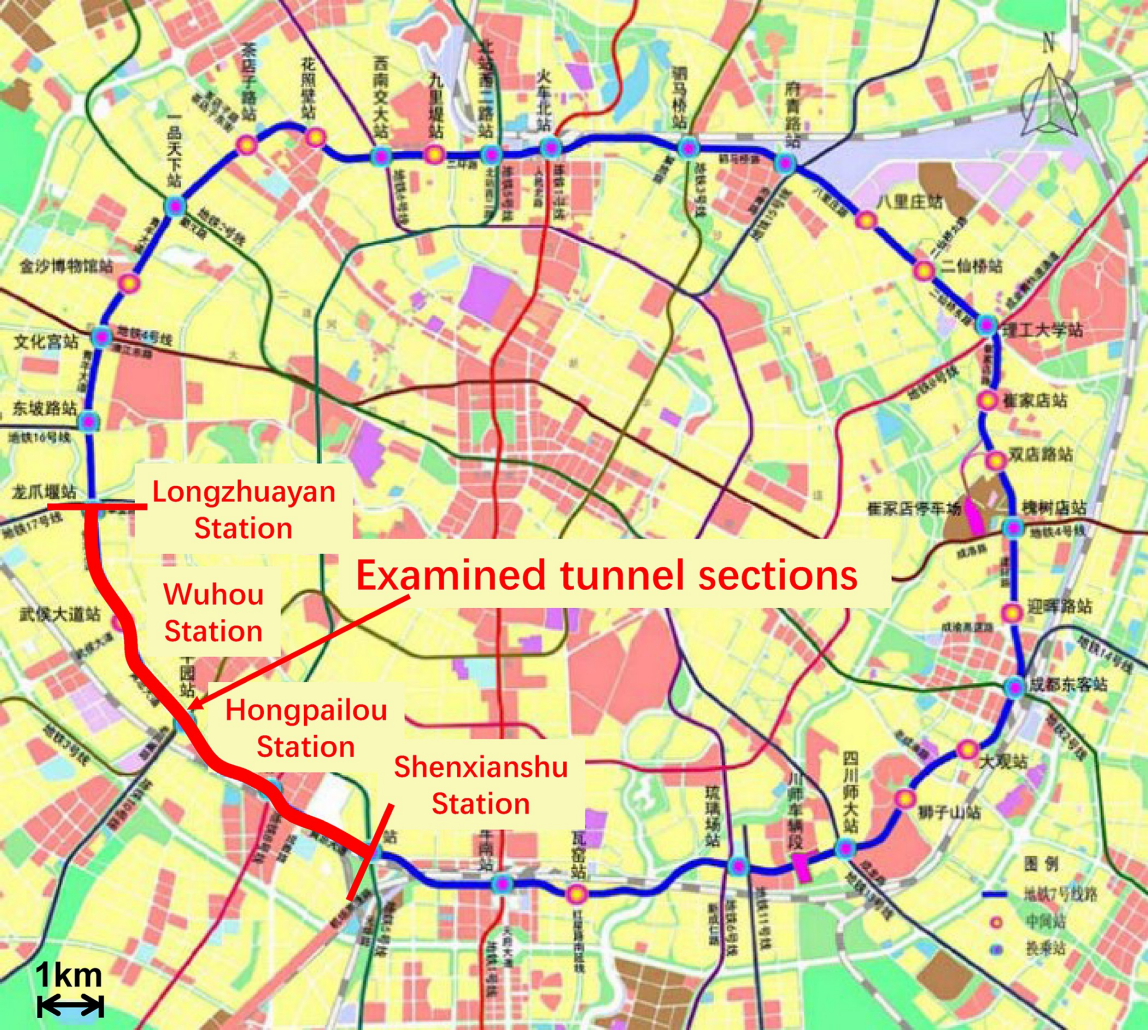
Locations of the examined tunnel sections.

The field data is collected from the shield tunneling of Chengdu Metro line 7. Six tunnels connecting four metro stations (with a total advancement of 9.4 km) are examined to obtain majority types of operational data. These tunnel sections are located on the southwest of Chengdu city, as shown in Fig 6. To avoid the influence of underground metro stations, data from the first and last 50 m of each tunnel are excluded. The six tunnels are excavated in sandy gravels. The content of gravels and sandy particles is 55% and 45%, respectively. The SPT value of the stratum is 12. The UCS value of the gravel is about 70 MPa. The EPB shield machines used in the tunnel construction are manufactured by CREG (China Railway Engineering Equipment Group). The main parameters of this EPB machine are shown in Table 2.

**Table 2:** Main parameters of EPB shield machine.

Parameter	Value
Lining width	1500 mm
Diameter of cutterhead	6280 mm
Shell thickness	40 mm
Lining installation gap	40 mm
Rotation speed of cutterhead	0~3 rpm
Designing torque	5500 kN·m
Maximum speed of excavation	80 mm/min
Maximum thrust	36000 kN
Shield length	9130 mm
Total weight of shield machine	≈500 t
Maximum working pressure	5 bar

### 3.2. Operational parameters and settlement measurements

**Fig 7:**
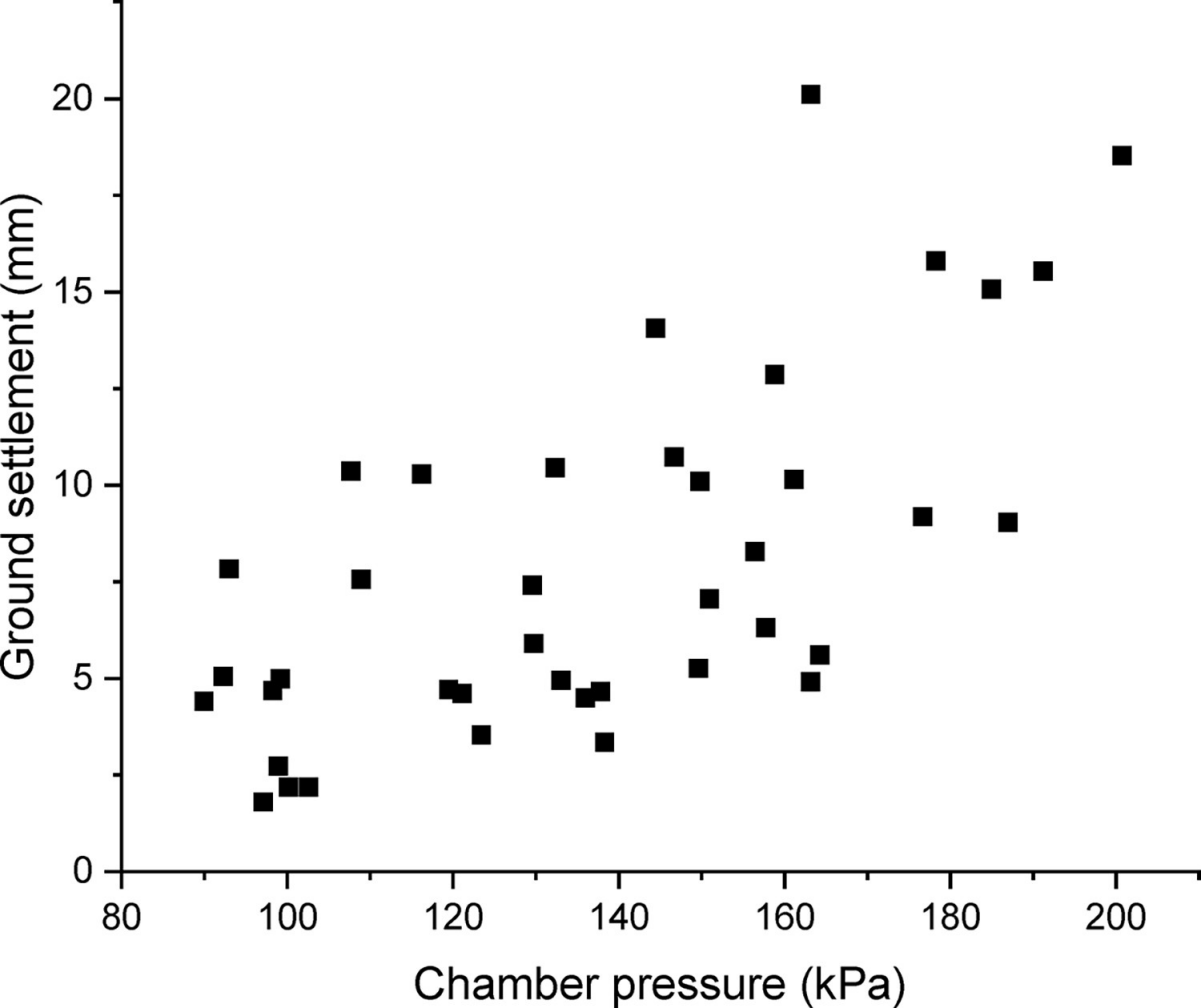
Relationship between the settlement and chamber pressure.

**Fig 8:**
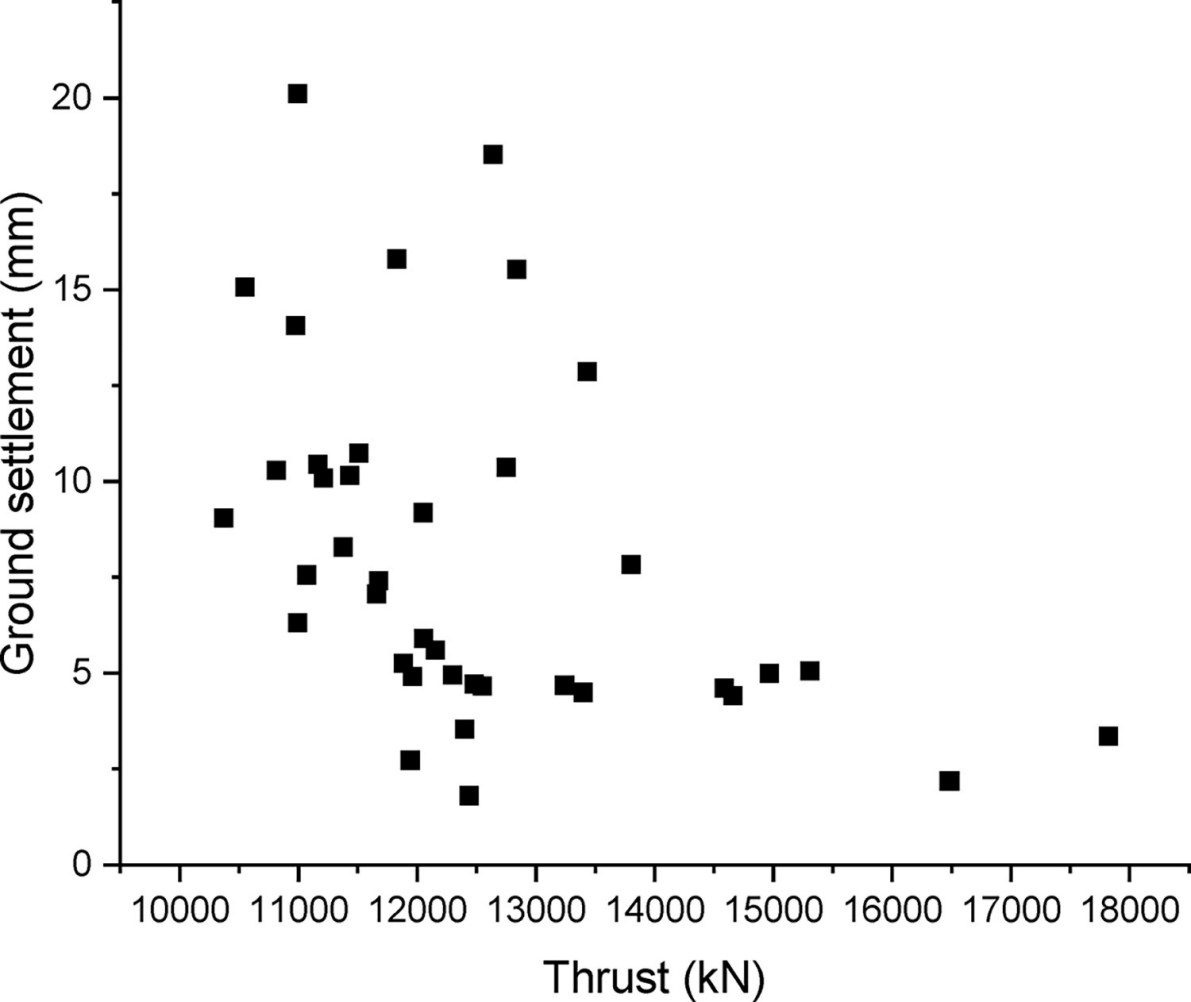
Relationship between the settlement and total thrust.

To eliminate the random fluctuations in operational parameters, average values of the operational data as well as the surface settlement are adopted every 100 rings (150 m). Such that the entire tunnels are divided into 40 separated segments. The relationship between measured settlement and main operational parameters, i.e. chamber pressure and tail thrust for each segment is shown in Figs 7 and 8. It should be noted that there were five earth pressure sensors located on the cutterhead, thus the chamber pressure was obtained from the average value of these five sensors. Because there is no significant difference in chamber pressure for the two stages, the chamber pressure shown in Fig 7 covers both the two stages during the excavation. Meanwhile, the thrust force shown in Fig 8 just covers the first stage in excavation. The summation of the force applied by four tail jacks was adopted as a total tail thrust. It can be observed that the measured settlement increases with the increase of chamber pressure. This trend is consistent with formula (15). However, without considering the effect of negative frictional force, it is difficult to explain this positive correlation between surface settlement and chamber pressure [2,19,20].

### 3.3. Validation of settlement prediction with frictional force

**Fig 9:**
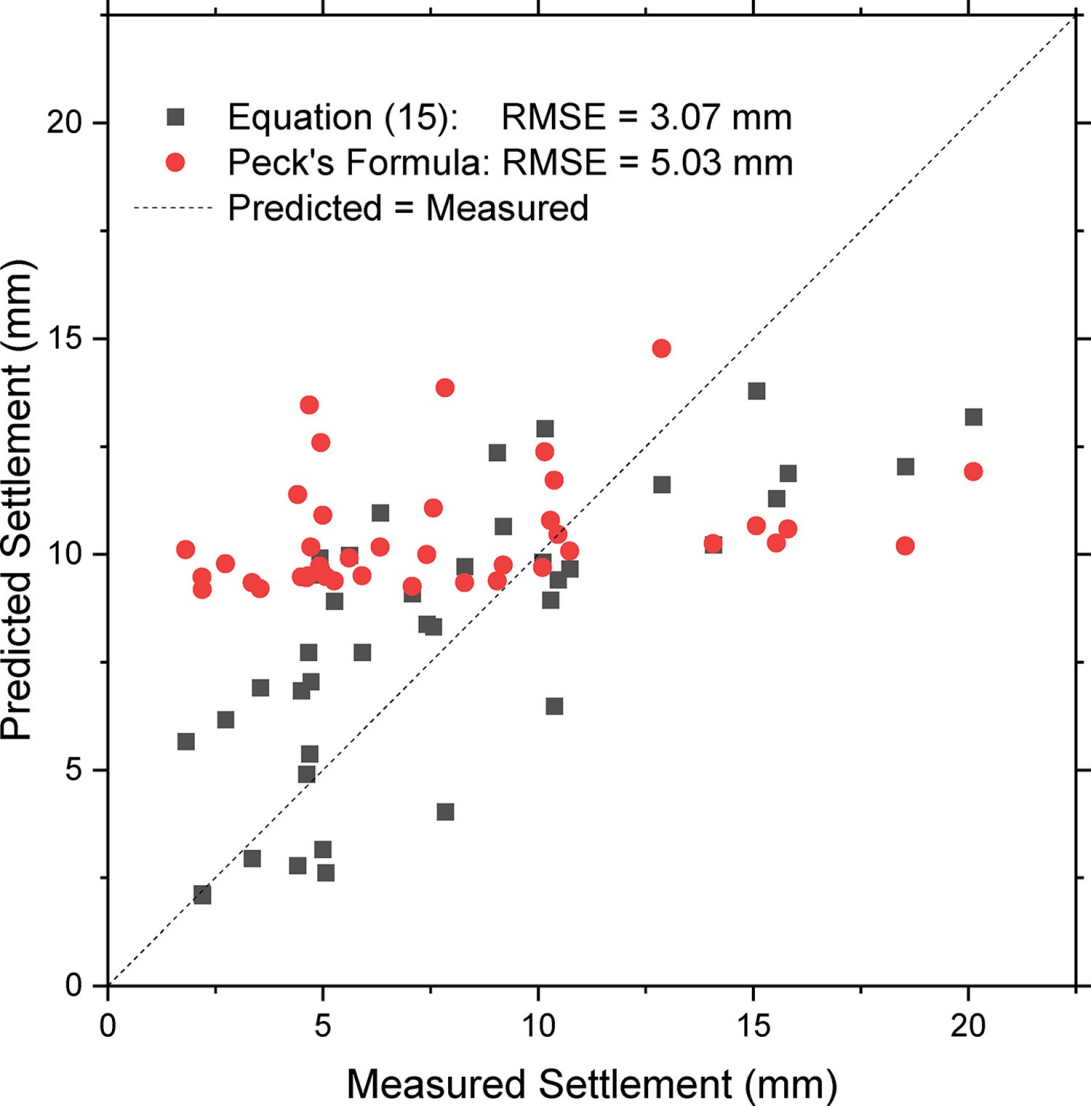
Performances of proposed model and Peck’s formula on 6 tunnels.

Considering the effect of negative frictional force, Eq (15) can be used to give quantitative prediction of surface settlement. This formula provides a comprehensive analysis of tunnel depth, operational parameters, and shield dimensions. Firstly, based on the shield length *L* = 9.13 m (see Table 2) and Poisson’s ratio *μ* = 0.25 (for sandy gravels), the parameters *a* and *b* in Eq (15) can be obtained. From Table 1, it can be determined that *a* = 13.7 m and *b* = 0.22. Then, with the sum of shell thickness and lining installation gap (both 40 mm as shown in Table 2), the width of tail gap *δ* can be obtained as 80 mm. Therefore, *V*_*loss*_ = 2π*Rδ* = 1.578 m^3^/m. Based on these parameters, as well as the real-time thrust *T* and chamber pressure *p*_*c*_, the surface settlement along all tunnel sections can be predicted by Eq (15). Fig 9 shows the comparison between the predicted and measured settlements. A good formula will produce scatters closer to the dashed diagonal, which is denoted by “predicted = measured” in Fig 9. It can be observed that the predicted values from Eq (15) are generally close to the dashed diagonal. The scatters from Eq (15) locate uniformly on both sides of the diagonal, thus there is no systematic bias. To evaluate the performance of Eq (15), the predicted settlements from Peck’s formula are also marked in Fig 9. In Peck’s formula, an empirical parameter of the ground volume loss ratio *V*_*lr*_ is used to predict the settlements. The parameter *V*_*lr*_ is not obtained from the physical volume of the tail gap. It is just determined empirically based on the classification of surrounding soils. For the sandy gravels in Chengdu district, it has been reported that the value of *V*_*lr*_ is 0.66%, and the width coefficient *k* is 0.39 [5]. With these empirical values, the surface settlement can be calculated based on the tunnel depth *h*. For the examined six tunnels, the central depth *h* varied between 14~23 m. Peck’s formula is very convenient to use, but it does not include any operational parameters or any physical processes. Therefore, it is impossible to consider the effects of operational parameters on surface settlement. As shown in Fig 9, the predicted surface settlement from Peck’s formula is limited in a narrow range of 9–15 mm, which is significantly different from the real distribution of measured settlements. Moreover, most of the scatter points of Peck’s formula (red circle) are located above the diagonal, indicating a systematic overestimation of predicted settlement. This systematic bias is induced by the limitation of input parameters in Peck’s formula. It is shown in Fig 9 that the prediction from Eq (15) is closer to the diagonal than the prediction from Peck’s formula. This indicates higher precision of the proposed formula. Consequently, the settlement calculated from Eq (15) is more reasonable than the results of Peck’s formula. The root mean square error (RMSE) of Eq (15) is 3.07 mm, which is 40% lower than the error of the Peck’s formula (RMSE = 5.03 mm). Eq (15) reflects the control of operational parameters on the surface settlement and thus can be used for optimizing the operational parameters (i.e. *p*_*c*_ and *T*). On the other hand, Peck’s formula has nothing to do with the operational parameters and it cannot provide useful information for the settlement control.

## 4. Discussion

The new model provides new insight in settlement control of EPB tunneling. Because of the negative frictional force, larger chamber pressure might result in larger surface settlement. Thus, the settlement control of EPB shield is based on the comprehensive relationship between thrust force and soil chamber pressure. The excavation control of EPB shield is not achievable by “earth pressure balance” itself. The fundamental reason for the negative frictional force is the complete unloading of tail jacks during the lining installation. To reduce the negative frictional force, the unloading time of tail jacks should be minimized. During the lining installation process, some thrust force might also be retained, which can avoid simultaneous unloading of all tail jacks.

It should be noted that the proposed model still ignores certain complexity of shield tunneling. For example, by using the Mindlin’s solution, the deformation of surrounding soil is supposed to be instant, which means the long-term creep is ignored. For the high permeability soils such as sandy gravels, the deformation converges rapidly, thus the influence of long-term creep is negligible. But the proposed model is not appropriate for clayey soils with slow deformation process. Another simplification in the model is that the grouting pressure is ignored. Because of the huge pores of sandy gravels, the grouting pressure varies so violently, it is difficult to obtain the effective value. Anyway, with the consideration of frictional force, the proposed formula provides more reasonable relationship between operational parameters and surface settlement in sandy gravels. It gives more useful prediction than traditional Peck’s formula, and it is also more convenient than existing integral formulas. Further research is needed to develop similar models for other types of ground soil.

## 5. Conclusions

During the EPB shield tunneling, the thrust force varies significantly at different stages. This results in negative frictional force at lining installation stage. A new formula is derived to evaluate the surface settlement induced by the frictional force at different stages. Conclusions were drawn as follows:

1. Traditionally, the shield tunneling was simplified as a uniform advancement. However, the actual EPB shield tunneling involves two distinct stages, i.e. advancement and lining installation. During the lining installation, the thrust of tail jacks is reduced to zero. This results in a complete reversal of the frictional force on the shield. The real frictional force can be calculated from the recorded chamber pressure and the tail thrust. For the EPB shield in sandy gravels of Chengdu district, the frictional force fluctuated between -26~30 kPa.
2. Based on the actual distribution of frictional force, a new formula of surface settlement was derived from Mindlin’s solution. The integral expression was then simplified to a closed form formula, which can provide fast estimation of the surface settlement with main operational parameters such as thrust and chamber pressure. In this new formula, the effect of frictional force at different stages is taken into account.
3. The proposed formula was validated against the field data of six EPB tunnels of Chengdu Metro. Field data indicated that tunnel-induced settlement in these sandy gravels increased with soil chamber pressure and decreased with jacking force. This observation was inconsistent with traditional settlement theories, but it was consistent with the new formula considering the effect of reversed friction. Moreover, the predictions of the proposed formula were in good agreement with the measured settlements. The RMSE of the proposed formula was 40% smaller than that of traditional Peck’s formula. With the real operational parameters, the proposed formula is also more useful than Peck’s formula for the control of excavation.
4. During the lining installation, increasing soil chamber pressure may lead to higher negative friction. Therefore, settlement control of EPB shield is based on comprehensive relationship between the soil chamber pressure and tail thrust. Generally, the unloading time of tail jacks should be minimized. During the lining installation, some thrust force might also be retained to avoid simultaneous unloading of all tail jacks.

It should be noted that the relationship between surface settlement and operational parameters is derived from Mindlin’s solution. The effect of creep deformation is ignored. The proposed model has been validated in sandy gravels with fast consolidation. Further researches are needed for EPB tunnels in clayey soils with slow deformation process.

## Supporting information

S1 Data (XLSX)

## References

[1] Xu Q, Lei S, Zhu Y, Liu Z, Zhang Z, Wang D, et al. Investigating the influence of excavating a tunnel undercrossing an existing tunnel at zero distance. PLoS One. 2024;19. doi: 10.1371/journal.pone.0301428 38625862 PMC11020911

[2] Farrokh E, Amiri A, Hasoomi A. Volume loss and face pressure evaluation in Tehran metro line 6, south extension. Tunnelling and Underground Space Technology. 2021;116. doi: 10.1016/j.tust.2021.104113

[3] Wang F, Du X, Li P. Predictions of ground surface settlement for shield tunnels in sandy cobble stratum based on stochastic medium theory and empirical formulas. Underground Space. 2023;11: 189–203.

[4] Peck RB. Deep excavations and tunneling in soft ground. Proceedings of 7th International Conference on Soil Mechanics and Foundation Engineering. Mexico City; 1969. pp. 225–290.

[5] Wu C, Zhu Z. Statistical analysis of ground loss ratio caused by different tunnel construction methods in China. Journal of Zhejiang University (Engineering Science). 2019;53: 19–30. doi: 10.3785/j.issn.1008-973X.2019.01.003

[6] Mair RJ. Settlement effects of bored tunnels. Proceedings of International Symposium on Geotechnical Aspects of Underground Construction in Soft Ground. London; 1996. pp. 43–53.

[7] Lu D, Lin Q, Tian Y, Du X, Gong Q. Formula for predicting ground settlement induced by tunnelling based on Gaussian function. Tunnelling and Underground Space Technology. 2020;103: 103443. doi: 10.1016/j.tust.2020.103443

[8] Ding Z, He SY, Zhou WH, Xu T, He SH, Zhang X. Analysis of ground deformation induced by shield tunneling considering the effects of muck discharge and grouting. Transportation Geotechnics. 2021;30. doi: 10.1016/j.trgeo.2021.100629

[9] Shi J, Wang F, Zhang D, Huang H. Refined 3D modelling of spatial-temporal distribution of excess pore water pressure induced by large diameter slurry shield tunneling. Comput Geotech. 2021;137. doi: 10.1016/j.compgeo.2021.104312

[10] Zhu C. Surface Settlement Analysis Induced by Shield Tunneling Construction in the Loess Region. Advances in Materials Science and Engineering. 2021;2021. doi: 10.1155/2021/5573372

[11] Zhu C, Wang S, Peng S, Song Y. Surface settlement in saturated loess stratum during shield construction: Numerical modeling and sensitivity analysis. Tunnelling and Underground Space Technology. 2022;119. doi: 10.1016/j.tust.2021.104205

[12] Liu Z, Ming W, Li J, Zhou C, Zhang L. Numerical prediction of the optimal shield tunneling strategy for tunnel construction in karst regions. PLoS One. 2021;16. doi: 10.1371/journal.pone.0252733 34086794 PMC8177454

[13] Zhang P, Wu HN, Chen RP, Dai T, Meng FY, Wang HB. A critical evaluation of machine learning and deep learning in shield-ground interaction prediction. Tunnelling and Underground Space Technology. 2020;106. doi: 10.1016/j.tust.2020.103593

[14] Su J, Wang Y, Niu X, Sha S, Yu J. Prediction of ground surface settlement by shield tunneling using XGBoost and Bayesian Optimization. Eng Appl Artif Intell. 2022;114: 105020. doi: 10.1016/j.engappai.2022.105020

[15] Ahangari K, Moeinossadat SR, Behnia D. Estimation of tunnelling-induced settlement by modern intelligent methods. Soils and Foundations. 2015;55: 737–748. doi: 10.1016/j.sandf.2015.06.006

[16] Chen RP, Zhang P, Kang X, Zhong ZQ, Liu Y, Wu HN. Prediction of maximum surface settlement caused by earth pressure balance (EPB) shield tunneling with ANN methods. Soils and Foundations. 2019;59: 284–295. doi: 10.1016/j.sandf.2018.11.005

[17] Suwansawat S, Einstein HH. Artificial neural networks for predicting the maximum surface settlement caused by EPB shield tunneling. Tunnelling and Underground Space Technology. 2006;21: 133–150. doi: 10.1016/j.tust.2005.06.007

[18] Suwansawat S, Einstein HH. Artificial neural networks for predicting the maximum surface settlement caused by EPB shield tunneling. Tunnelling and Underground Space Technology. 2006;21: 133–150. doi: 10.1016/j.tust.2005.06.007

[19] Society of foundation engineering. Investigation, design and construction of shield tunnels. Tokyo: Maruzen; 1997.

[20] Li Z, Chen R, Meng F, Ye J. Tunnel boring machine tunneling-induced ground settlements in soft clay and influence of excavation parameters. Journal of Zhejiang University (Engineering Science). 2015;49: 1268–1275.

[21] Guo S, Wang B, Zhang P, Wang S, Guo Z, Hou X. Influence analysis and relationship evolution between construction parameters and ground settlements induced by shield tunneling under soil-rock mixed-face conditions. Tunnelling and Underground Space Technology. 2023;134: 105020. doi: 10.1016/j.tust.2023.105020

[22] Zhang X, Luo B, Xu Y, Yang Z. Theoretical analysis of stratum horizontal displacements caused by small radius curve shield tunneling. Comput Geotech. 2024;165: 105950. 10.1016/j.compgeo.2023.105950.

[23] Cao L, Chen X, Lu D, Zhang D, Su D. Theoretical prediction of ground settlements due to shield tunneling in multi-layered soils considering process parameters. Underground Space. 2024;16: 29–43. 10.1016/j.undsp.2023.07.007.

[24] Xin L, Chen LG, Li T, Yu YL, Wu B. Calculation of additional load and deformation of the receiving well enclosure structure caused by shield tunneling. PLoS One. 2024;19. doi: 10.1371/journal.pone.0297912 38573995 PMC10994317

[25] Yang XL, Wang JM. Ground movement prediction for tunnels using simplified procedure. Tunnelling and Underground Space Technology. 2011;26: 462–471. 10.1016/j.tust.2011.01.002.

[26] Yang JS, Liu BC, Wang MC. Modeling of tunneling-induced ground surface movements using stochastic medium theory. Tunnelling and Underground Space Technology. 2004;19: 113–123. 10.1016/j.tust.2003.07.002.

[27] Wei G, Zhang S, Qi J, Yao N. Study on calculation method of ground deformation induced by shield tunnel construction. Chinese Journal of Rock Mechanics and Engineering. 2006; 3317–3323.

[28] Wei G, Xu R. Prediction of longitudinal ground deformation due to tunnel construction with shield in soft soil. Chinese Journal of Geotechnical Engineering. 2005; 1077–1081.

[29] Sagaseta C. Analysis of undrained soil deformation due to ground loss. Géotechnique. 1987;37: 301–320.

[30] Xiaowu Tang, Ji Zhu, Wei Liu, Renpeng Chen. Research on soil deformation during shield construction process. Chinese Journal of Rock Mechanics and Engineering. 2010;29: 417–422.

[31] Gou B. Research on surface deformation prediction method of shield tunnel. Beijing University of Technology. 2022.

